# Dual Proteasome and Histone Deacetylase Inhibition Overcomes Tyrosine Kinase Inhibitor Resistance in Breakpoint Cluster Region: Abelson 1‐Driven Leukaemia Cell Lines

**DOI:** 10.1111/jcmm.71053

**Published:** 2026-02-16

**Authors:** Seiichi Okabe, Seiichiro Yoshizawa, Yuya Arai, Akihiko Gotoh, Daigo Akahane

**Affiliations:** ^1^ Department of Hematology Tokyo Medical University Tokyo Japan

**Keywords:** apoptosis, asciminib resistance, BCR::ABL1, bortezomib, CML, HDAC, panobinostat, proteostasis

## Abstract

Resistance to tyrosine kinase inhibitors (TKIs) remains a major challenge in breakpoint cluster region (BCR)::Abelson 1 (ABL1)‐driven leukaemias. Asciminib offers a novel therapeutic option; however, resistance continues to emerge. We hypothesised that targeting proteostasis and epigenetic regulation with bortezomib and panobinostat could eliminate TKI‐refractory cells via TKI‐independent mechanisms. We profiled parental and TKI‐resistant chronic myelogenous leukaemia (CML) and Ba/F3 models. Viability, cytotoxicity, and caspase‐3/7 activity were assessed following single‐agent treatment with asciminib, ponatinib, bortezomib, or panobinostat. The effects of the bortezomib–panobinostat combination on colony formation, mitochondrial membrane potential, and apoptosis were evaluated. Asciminib showed reduced potency in resistant models and a right‐shifted dose–response curve in T315I cells, whereas ponatinib retained activity across BCR::ABL1 variants. Bortezomib and panobinostat induced low‐nanomolar cytotoxicity and robust caspase‐3/7 activation in resistant lines. The combination of bortezomib and panobinostat showed modest trends toward reduced cell viability and increased cytotoxicity and caspase‐3/7 activity, especially in TKI‐resistant cells. The combination suppressed clonogenic growth and triggered apoptosis in resistant cells. Co‐inhibition of proteasomes and histone deacetylases eliminates TKI‐refractory BCR::ABL1‐driven leukaemia cells by inducing mitochondrial apoptosis and loss of clonogenic potential. These findings indicate a clinically actionable, TKI‐independent strategy for the salvage treatment of multidrug‐resistant CML.

## 1 Introduction

Chronic myeloid leukaemia (CML) is caused by BCR::ABL1 tyrosine kinase, which through its continuous activity, triggers and sustains leukemogenesis [1, 2]. While tyrosine kinase inhibitors (TKIs) targeting BCR::ABL1 have improved CML prognosis, resistance and intolerance remain significant clinical challenges [1]. Resistance arises from kinase domain mutations that hinder drug binding, amplification or structural changes in BCR::ABL1, and kinase‐independent adaptations that maintain survival signalling [1]. Among resistant lesions, the T315I gatekeeper mutation is particularly challenging, as it confers high‐level resistance to first‐ and second‐generation ATP‐competitive TKIs [3]. Two complementary third‐line approaches have been developed: ponatinib, a potent ATP‐competitive inhibitor effective against most single mutations including T315I, and asciminib, a pioneering allosteric STAMP (Specifically Targeting the ABL Myristoyl Pocket) inhibitor that binds the myristoyl pocket to stabilise BCR::ABL1 in an inactive conformation [4]. Despite these advances, some patients develop refractory disease characterised by compound mutations, clonal selection with unique vulnerabilities, or reprogramming of apoptotic pathways, ultimately leading to disease progression with few remaining treatment options [5]. This highlights the need for broadly active therapeutic strategies that remain effective despite BCR::ABL1 alterations or TKI failure, emphasising the importance of understanding signalling network rewiring in resistant cells [6].

Targeting oncogene dependency has transformed the treatment of several cancers and remains a powerful therapeutic approach. However, alternative strategies are needed for cancers driven by undruggable oncogenes, loss of tumour suppressor genes, or those that develop resistance despite reliance on targetable drivers [7]. The ubiquitin–proteasome system (UPS), the principal pathway for intracellular protein degradation, is essential for regulating the stability of key proteins involved in cancer progression, particularly in hematologic malignancies [8]. Proteasome inhibition, which targets the complex responsible for degrading ubiquitinated proteins, has emerged as an effective strategy in hematologic malignancies, notably multiple myeloma [9].

Histone deacetylase (HDAC) inhibitors, such as panobinostat, represent a relatively new class of anticancer agents that play critical roles in both epigenetic and non‐epigenetic regulation. They induce cancer cell death, trigger apoptosis, and cause cell cycle arrest [10]. HDACs are crucial in haematopoiesis, influencing both normal and malignant states, and their dysregulated expression is linked to the initiation, progression, and recurrence of leukaemia, lymphoma, and multiple myeloma [11]. HDAC inhibitors can activate apoptosis via both the intrinsic (mitochondrial) and extrinsic (death receptor) pathways [12].

Based on these considerations, we investigated whether proteasomal and HDAC inhibition could overcome resistance to ABL‐directed TKIs and provide additional benefits across diverse CML models. Using asciminib and ponatinib as benchmarks, we compared parental and resistant K562 and Ba/F3 cell lines, including those expressing BCR::ABL1 wild‐type or T315I mutant. We further assessed the effects of bortezomib and panobinostat, individually and in combination, on cell viability, cytotoxicity, caspase‐3/7 activation, mitochondrial membrane potential, and colony formation. This comprehensive approach allowed us to link immediate drug responses to prolonged clonogenic inhibition, which is closely associated with relapse risk.

## 2 Materials and Methods

### 2.1 Cell Lines and Culture

The CML cell line K562 was obtained from the American Type Culture Collection (ATCC; Manassas, VA, USA). Wild‐type BCR::ABL1–expressing Ba/F3 cells (Ba/F3 BCR::ABL), Ba/F3 T315I cells harbouring the BCR::ABL1 T315I mutation, and asciminib‐resistant Ba/F3 cells (Ba/F3 asc‐R) were established as previously described [13, 14]. In addition, previous studies have generated and characterised ponatinib‐resistant (K562 PR) and imatinib‐resistant (K562 IR) derivatives of K562 [15, 16].

All cell lines were maintained in RPMI 1640 medium supplemented with 10% fetal calf serum, 1% penicillin–streptomycin, and cultured at 37°C in a humidified atmosphere with 5% CO_2_. Ba/F3 cell lines were additionally supplemented with 10% WEHI‐3–conditioned medium as a source of interleukin‐3.

### 2.2 Reagents

Ponatinib, bortezomib, and panobinostat were purchased from Selleck Chemicals (Houston, TX, USA) and dissolved in dimethyl sulfoxide (DMSO) to prepare 10 mM stock solutions. Asciminib was obtained from Active Biochem (Baileys Harbour, WI, USA). All compounds were stored at −80°C and thawed only once before use. The final DMSO concentration in all experiments was kept below 0.1% (v/v). Unless otherwise specified, additional reagents were sourced from Merck KGaA (Darmstadt, Germany).

### 2.3 Drug Screening

Drug screening was performed using 96‐well clear, round, flat‐bottom plates. Each test compound was diluted in DMSO to 2 mM and dispensed into wells to achieve a final concentration of 1 μM (final volume, 0.05 μL). A total of 1900 mechanistically annotated compounds were evaluated. Ba/F3 asc‐R cells (4 × 10^3^ cells per well) were plated and cultured for 72 h at 37°C in a humidified atmosphere containing 5% CO₂. Cell viability was then determined using the Cell Counting Kit‐8 (Dojindo Laboratories, Kumamoto, Japan). Compounds that significantly reduced viability relative to DMSO‐treated controls were selected as potential candidates.

### 2.4 Drug Exposure Schemes

For single‐agent dose–response experiments, cells were treated with 10‐fold serial dilutions ranging from 0 to 1 μM for asciminib and ponatinib and from 0 to 100 nM for bortezomib and panobinostat. For fixed‐dose combination experiments, bortezomib and panobinostat were each used at 10 nM, unless otherwise specified. Vehicle controls (0.1% DMSO) were included in each plate and matched across all dilution series.

### 2.5 Cell Viability Assay

Cells were plated in clear, flat‐bottom 96‐well plates (K562: 5 × 10^3^ cells/well; Ba/F3: 4 × 10^3^ cells/well; total volume = 100 μL/well) and treated as described above. After 72 h of incubation, cell viability was assessed using the Cell Counting Kit‐8 (CCK‐8; Dojindo Laboratories) according to the manufacturer's instructions. CCK‐8 reagent (10 μL per well, 1:10 v/v) was added, and plates were incubated for 1–3 h at 37°C until sufficient colour development occurred. Absorbance was measured at 450 nm, with 600–650 nm as the reference wavelength, using a Nivo Multimode Plate Reader (PerkinElmer, Waltham, MA, USA). The combined effects of bortezomib and panobinostat were evaluated using the Chou–Talalay method. Combination index (CI) values were calculated using CompuSyn software (ComboSyn Inc., Paramus, NJ, USA), where CI < 1, CI = 1, and CI > 1 indicate synergistic, additive, and antagonistic effects, respectively [17].

### 2.6 Cytotoxicity Assay

CML cells were exposed to varying doses of asciminib, ponatinib, bortezomib, and/or panobinostat. Following a 48‐h treatment, cytotoxicity was measured by lactate dehydrogenase (LDH) release using the Cytotoxicity LDH Assay Kit (Dojindo Laboratories) per the manufacturer's instructions. Absorbance was read on a Nivo Multimode Plate Reader (PerkinElmer).

### 2.7 Caspase‐3/7 Activity

Caspase‐3/7 activity in CML cells was assessed using the Caspase‐Glo 3/7 Assay Kit (Promega, Madison, WI, USA) according to the manufacturer's protocol. Cells were treated with different concentrations of bortezomib, panobinostat, or their combination. After 48 h, an equal volume of Caspase‐Glo 3/7 reagent was added to each well, and luminescence was recorded using a Nivo Multimode Plate Reader.

### 2.8 Mitochondrial Membrane Potential (ΔΨm)

Cells were treated with bortezomib and/or panobinostat for 48 h. Mitochondrial membrane potential (ΔΨm) was assessed using the JC‐1 MitoMP Detection Kit (Dojindo Laboratories) following the manufacturer's protocol. Cells were stained with 2 μM JC‐1 dye for 20 min at 37°C, washed, and analysed for fluorescence using a Nivo Multimode Plate Reader (PerkinElmer) at excitation/emission wavelengths of 485/535 nm (green) and 535/590 nm (red). The red‐to‐green (R/G) fluorescence ratio was calculated, with a reduction indicating mitochondrial depolarisation.

### 2.9 Flow Cytometric Analysis of Apoptosis

Cells were treated with bortezomib and/or panobinostat for 24 h. After washing with cold phosphate‐buffered saline, cells were resuspended in Annexin V binding buffer and stained with Annexin V–FITC and propidium iodide (PI) for 15 min at room temperature in the dark (Annexin V–FITC Apoptosis Detection Kit, Nacalai Tesque, Kyoto, Japan). Samples were analysed within 1 h using a benchtop flow cytometer (BD Biosciences, San Jose, CA, USA), recording a minimum of 10,000 events per sample. Gates were set using unstained and single‐stained controls. Early apoptotic (Annexin V^+^/PI^−^) and late apoptotic (Annexin V^+^/PI^+^) populations were combined and reported as a percentage of total events.

### 2.10 Colony Formation Assay

Colony formation was assessed using MethoCult Express (Catalog #04437; STEMCELL Technologies, Vancouver, BC, Canada), a methylcellulose‐based medium, following the manufacturer's instructions. Briefly, 1–3 × 10^2^ K562 cells were mixed into MethoCult Express medium with specified concentrations of bortezomib and/or panobinostat. Cultures were incubated at 37°C in a humidified incubator with 5% CO_2_ for 7 days. Colonies were counted, and representative images were captured using an EVOS FL Digital Inverted Fluorescence Microscope (Thermo Fisher Scientific, Waltham, MA, USA). All experiments were performed in triplicate, and results are presented as means ± standard errors of the mean.

### 2.11 Statistical Analyses

Statistical analyses were conducted using GraphPad Prism version 10 (GraphPad Software, San Diego, CA, USA). Data are expressed as means ± standard deviations (SDs) from at least three independent experiments. Comparisons between two groups were analysed using the paired Student's *t*‐test. For multiple group comparisons, one‐way or two‐way analysis of variance (ANOVA) followed by Dunnett's post hoc test was used, with an alpha level of 0.05. Statistical significance was indicated as *p* < 0.05, *p* < 0.01, *p* < 0.001, and *p* < 0.0001.

## 3 Results

### 3.1 Differential Sensitivity of CML Cell Lines to Asciminib and Ponatinib

**FIGURE 1:**
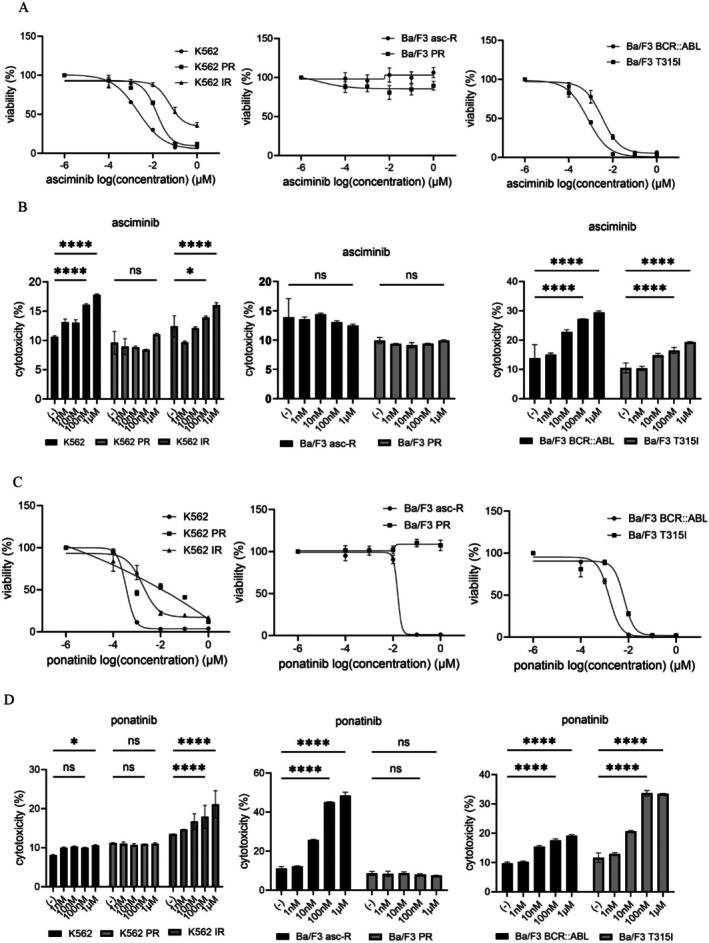
Responses to asciminib and ponatinib in parental and resistant chronic myelogenous leukaemia (CML) models. (A) CML cell lines (K562, K562 PR, K562 IR, Ba/F3 asc‐R, Ba/F3 PR, Ba/F3 BCR::ABL, and Ba/F3 T315I) were exposed to asciminib (0–1 μM) for 72 h, and cell viability was measured using the Cell Counting Kit‐8 (CCK‐8) assay. (B) The same CML cell lines were treated with asciminib (0–1 μM) for 48 h, and cytotoxicity was assessed using a Cytotoxicity LDH Assay Kit. Data were normalised to untreated controls and expressed as means ± SD. *p < 0.05, ****p < 0.0001 vs. control; ns, not significant. (C) Ponatinib (0–1 μM) was administered to the CML cells for 72 h, and cell viability was measured using the CCK‐8 assay. (D) Cytotoxicity in CML cells treated with ponatinib (0–1 μM) for 48 h was determined using the LDH assay. Results were normalised to controls and presented as means ± SD. *p < 0.05, ****p < 0.0001 vs. control; ns, not significant.

Asciminib is a STAMP inhibitor approved for CML [18]. We evaluated the therapeutic efficacy of asciminib and ponatinib in cell lines resistant to ABL TKIs. Asciminib reduced cell viability in parental K562 and Ba/F3 BCR::ABL1 cells but exhibited partial effects on imatinib‐ and ponatinib‐resistant cells and minimal effects on asciminib‐resistant cells (Figure 1A). Asciminib‐induced cytotoxicity, assessed by LDH release, was significantly lower in resistant cell lines compared with parental cells (Figure 1B). In contrast, ponatinib markedly decreased cell viability in parental, T315I‐mutant, and asciminib‐resistant cells (Figure 1C). LDH assays confirmed that ponatinib induced a dose‐dependent increase in cytotoxicity in parental and T315I cells, but not in ponatinib‐resistant cells (Figure 1D). The limited apparent effect of ponatinib in wild‐type K562 cells is likely attributable to early cytotoxic saturation due to high intrinsic sensitivity under experimental conditions. These findings indicate that asciminib‐resistant cells retain responsiveness to ponatinib, similar to T315I‐mutant CML cells.

### 3.2 Identification of Bortezomib and Panobinostat by Drug Screening and Evaluation of Bortezomib Efficacy in CML Cells

**FIGURE 2:**
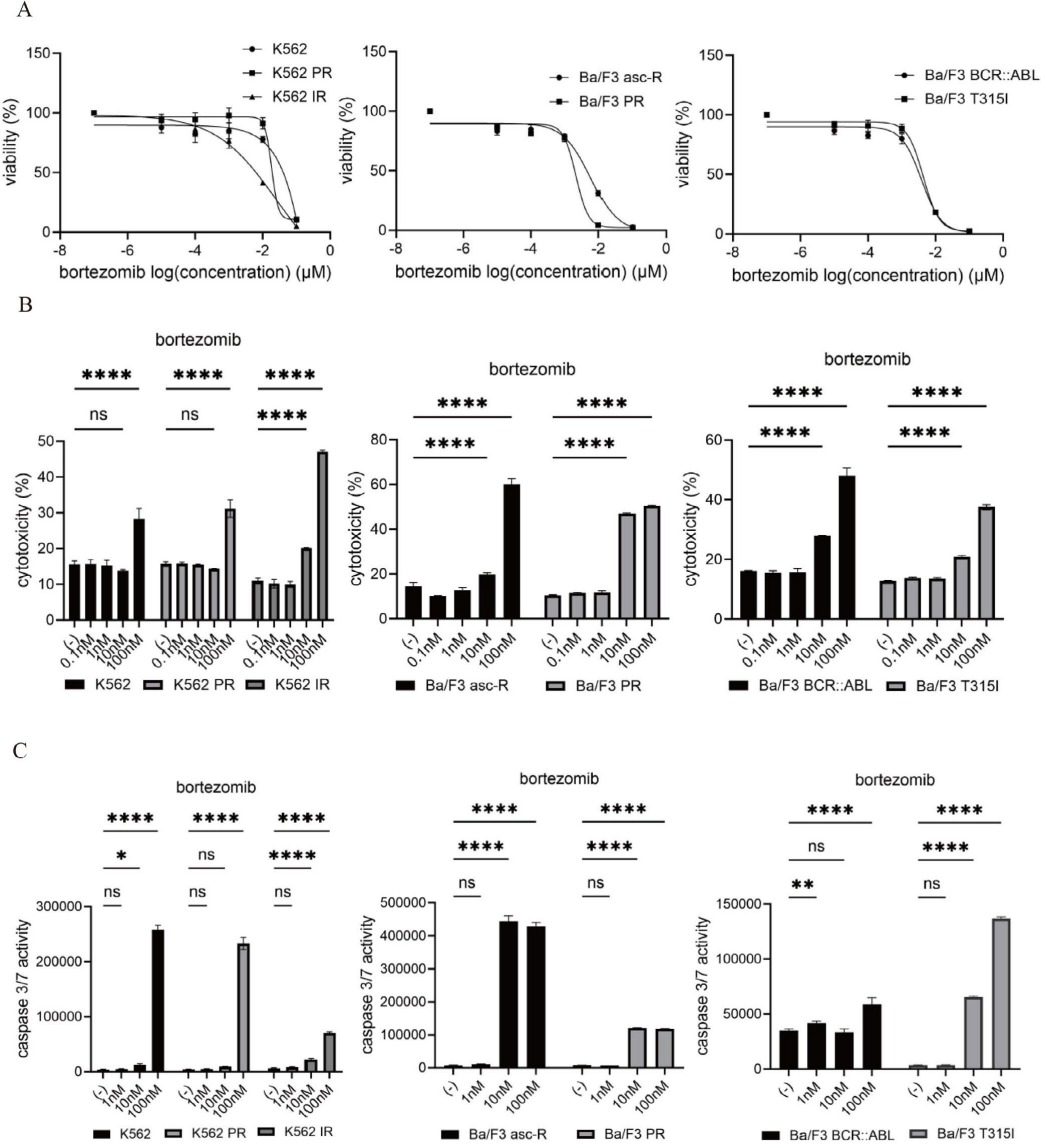
Proteasome inhibitor bortezomib suppresses growth and induces apoptosis in parental and TKI‐resistant CML models. (A) CML cell lines (K562, K562 PR, K562 IR, Ba/F3 asc‐R, Ba/F3 PR, Ba/F3 BCR::ABL, and Ba/F3 T315I) were exposed to bortezomib (0–100 nM) for 72 h, and cell viability was determined using the CCK‐8 assay. (B) Cytotoxicity was measured in the same cell lines following 48‐h exposure to bortezomib (0–100 nM) using the LDH assay. Data were normalised to untreated controls and expressed as means ± SD. *p < 0.05, ****p < 0.0001 vs. control; ns, not significant. (C) Caspase‐3/7 activity was measured in CML cells treated with bortezomib (0–100 nM) for 48 h. *p < 0.05, ****p < 0.0001 vs. control; ns, not significant.

Next, we performed drug screening using the Ba/F3 asc‐R cell line. Among the 1900 screened compounds, bortezomib, a proteasome inhibitor, and panobinostat emerged as promising candidates (data not shown). Then, we evaluated the efficacy of bortezomib in ABL TKI‐resistant cells. Bortezomib reduced the viability of both parental and resistant CML cell lines, including Ba/F3 asc‐R and K562 PR cells (Figure 2A). Cytotoxic effects were observed in parental, T315I‐mutant, and resistant cells and increased in a dose‐dependent manner across all lines (Figure 2B). Caspase‐3/7 assays confirmed that bortezomib induced apoptosis in both sensitive and resistant cell lines (Figure 2C). These results suggest that bortezomib retains potent cytotoxic activity and may overcome TKI resistance in CML cells.

### 3.3 Panobinostat Effectively Induces Cytotoxicity and Apoptosis in Both TKI‐Sensitive and TKI‐Resistant CML Cells

**FIGURE 3:**
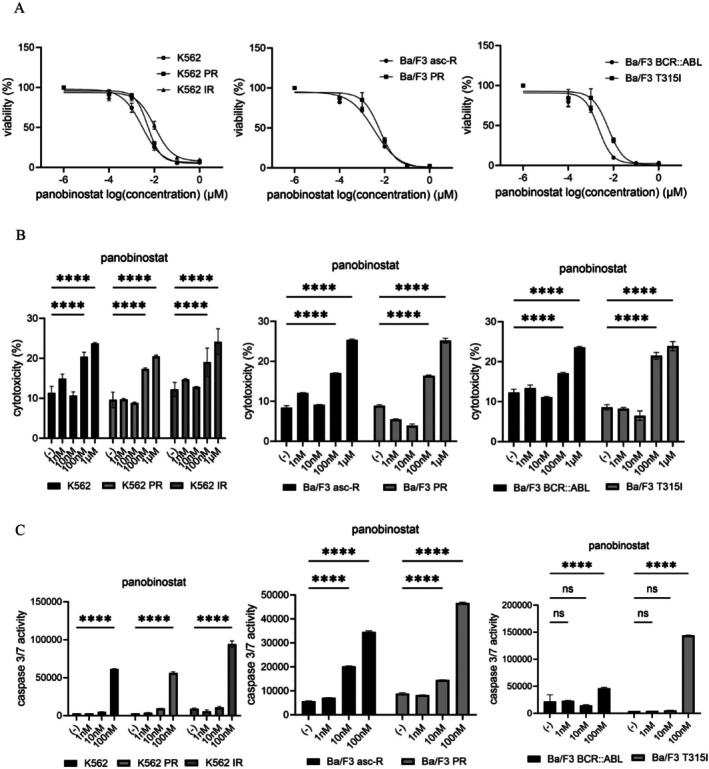
Panobinostat exhibits broad, dose‐dependent antileukemic activity in parental and drug‐resistant CML models. (A) CML cell lines (K562, K562 PR, K562 IR, Ba/F3 asc‐R, Ba/F3 PR, Ba/F3 BCR::ABL, and Ba/F3 T315I) were treated with panobinostat (0–100 nM) for 72 h, and cell viability was assessed using the CCK‐8 assay. (B) Cytotoxicity following 48‐h exposure to panobinostat (0–100 nM) was measured using the LDH assay. Data are expressed as means ± SD. ****p < 0.0001 vs. control. (C) Caspase‐3/7 activity was evaluated in CML cells treated with panobinostat (0–100 nM) for 48 h. ****p < 0.0001 vs. control; ns, not significant.

Panobinostat, an HDAC inhibitor originally approved for relapsed or refractory multiple myeloma [19], was evaluated in CML cells. Panobinostat reduced cell viability in both parental and resistant cell lines, including Ba/F3 asc‐R and K562 PR cells (Figure 3A). Cytotoxicity increased in a dose‐dependent manner in all lines, with more pronounced effects in parental and T315I‐mutant cells (Figure 3B). Resistant cells retained partial sensitivity to panobinostat. Caspase‐3/7 activity assays confirmed that panobinostat induced apoptosis in both sensitive and resistant cells (Figure 3C). These findings indicate that panobinostat effectively triggers apoptosis in CML cells resistant to TKIs.

### 3.4 Combination Treatment With Bortezomib and Panobinostat Enhances Cytotoxicity in CML Cells

**FIGURE 4:**
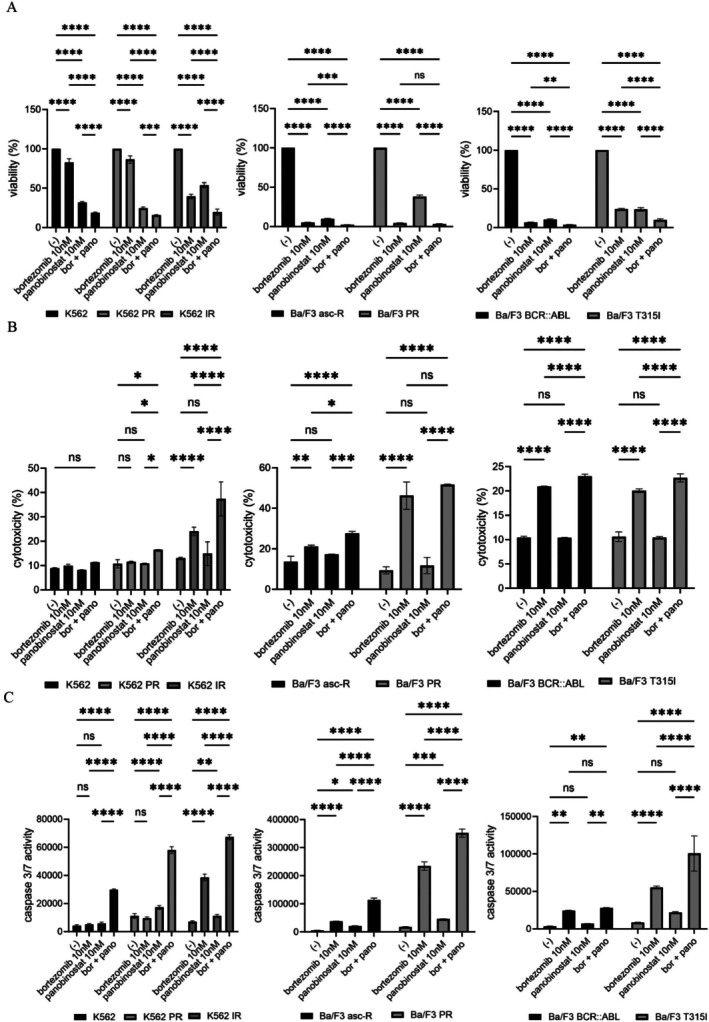
Combined bortezomib and panobinostat treatment enhances antileukemic activity in CML models. (A–C) CML cell lines (K562, K562 PR, K562 IR, Ba/F3 asc‐R, Ba/F3 PR, Ba/F3 BCR::ABL, and Ba/F3 T315I) were treated with 10 nM bortezomib and/or 10 nM panobinostat for 48 or 72 h. (A) Cell viability, (B) cytotoxicity, and (C) caspase‐3/7 activity were assessed. *p < 0.05, **p < 0.01, ***p < 0.001, ****p < 0.0001 vs. control; ns, not significant.

Subsequently, we examined the effects of combined bortezomib and panobinostat treatment in CML cell lines, including ABL TKI‐resistant models. The combination significantly decreased cell viability in both parental and resistant cells (Figure 4A) and induced greater cytotoxicity than either agent alone, except in K562 cells (Figure 4B). In contrast, Ba/F3 cells were not inhibited (Supplemental Figure 1). Furthermore, the Chou–Talalay method indicated that the CI was less than 1, suggesting synergistic interactions between bortezomib and panobinostat. The strongest effects were observed in parental and T315I‐mutant cells, while resistant cells showed partial sensitivity. Caspase‐3/7 assays demonstrated a marked increase in apoptosis with combination treatment (Figure 4C). These results suggest that bortezomib and panobinostat act synergistically to induce antileukemic effects and may overcome drug resistance in CML cells.

### 3.5 Combined Treatment With Bortezomib and Panobinostat Suppresses Colony Formation and Induces Apoptosis in CML Cells

**FIGURE 5:**
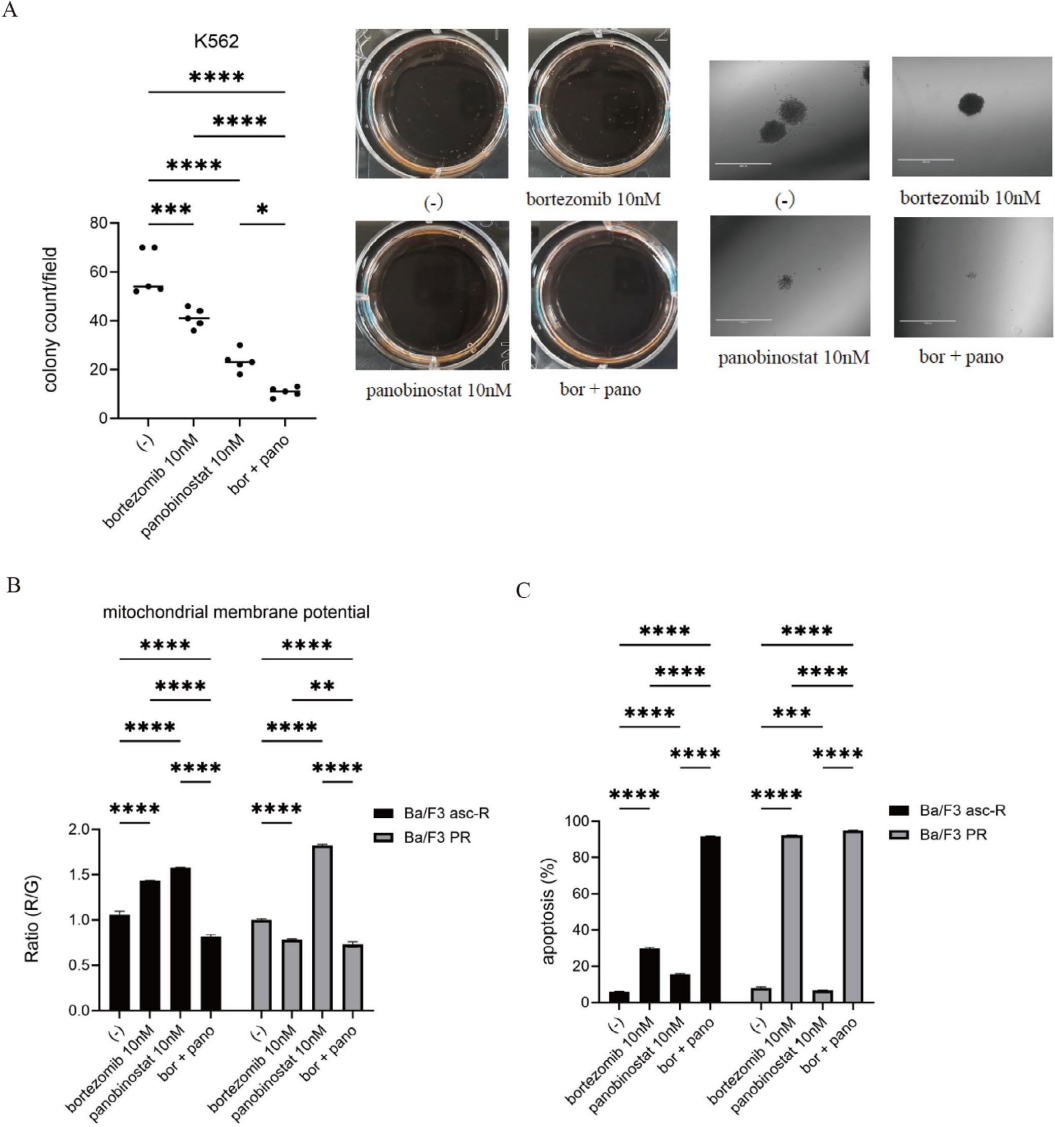
Bortezomib plus panobinostat suppresses clonogenicity, disrupts mitochondrial membrane potential, and induces apoptosis. (A) K562 cells were exposed to 10 nM bortezomib, with or without 10 nM panobinostat, for 7–9 days. Colonies were photographed using a digital microscope and counted. Representative images and quantification from three independent experiments are shown (scale bar: 1000 μm). Statistical significance: ***p < 0.001, ****p < 0.0001 vs. control. (B) Ba/F3 asc‐R and Ba/F3 PR cells were treated with bortezomib ± panobinostat for 48 h, and mitochondrial membrane potential was assessed using a mitochondrial staining kit. ****p < 0.0001 vs. control. (C) Ba/F3 asc‐R and Ba/F3 PR cells were treated with bortezomib ± panobinostat for 24 h. Apoptosis assays showed minimal induction with single agents, whereas the combination markedly increased apoptotic cell levels. Statistical significance: ***p < 0.001, ****p < 0.0001 vs. control.

Finally, we assessed the impact of these agents on colony formation. The combination of bortezomib and panobinostat led to a pronounced reduction in both colony number and size compared with either drug alone (Figure 5A). Microscopic examination confirmed marked inhibition of colony growth in the combination group. Furthermore, mitochondrial membrane potential was significantly reduced following combined treatment, indicating mitochondrial dysfunction (Figure 5B). Flow cytometry revealed a substantial increase in apoptotic cells compared with single‐agent treatments (Figure 5C). These results demonstrate that bortezomib and panobinostat synergistically induce mitochondrial dysfunction and apoptosis, effectively suppressing CML cell growth.

## 4 Discussion

This research illustrates that the combination of proteasome and HDAC inhibitors effectively impairs the survival of CML cells and addresses resistance to ABL TKIs. While asciminib and ponatinib remain pivotal in CML treatment, resistance to ABL TKIs—particularly in cells with compound mutations or those exhibiting adaptive signalling—continues to challenge long‐term efficacy. Our findings indicate that bortezomib and panobinostat maintain strong cytotoxic and pro‐apoptotic effects in both parental and resistant CML models, including cells unresponsive to asciminib or ponatinib.

The mechanisms underlying TKI resistance can be broadly classified as BCR::ABL1‐dependent or BCR::ABL1‐independent. BCR::ABL1‐dependent resistance primarily involves mutations within the kinase domain that reduce TKI binding affinity, whereas BCR::ABL1‐independent resistance arises from factors such as increased drug efflux, miRNA dysregulation, and activation of alternative signalling pathways [20].

In patients with Philadelphia chromosome‐positive acute lymphoblastic leukaemia and advanced CML, compound mutations frequently arise. These mutations involve multiple alterations within the same BCR::ABL1 molecule in a single leukaemic cell, often conferring substantial or complete resistance to individual TKIs [21]. Some compound mutations remain partially responsive to high concentrations of ponatinib, the most potent TKI available; however, its dose‐dependent toxicity necessitates careful adjustment and optimisation of dosing strategies [21]. Compound mutations involving the gatekeeper T315I mutation—one of the most common—typically result in complete resistance to all currently approved single TKIs, including ponatinib and asciminib [21]. Among patients with CML receiving TKI therapy, approximately 70% of double mutations detected in the BCR::ABL1 kinase domain by direct sequencing are identified as compound mutations [5].

Ubiquitination plays a crucial role in regulating complex cellular processes, including protein degradation, protein–protein interactions, endocytosis, cell‐cycle progression, and substrate activation or inactivation [22]. Cancer cells exhibit rapid protein turnover and an increased reliance on maintaining proteostasis compared with normal cells. Accordingly, the UPS is critical for their survival and represents an attractive target for anticancer drug development [23]. In CML, UPS components can modulate TKI efficacy by regulating BCR::ABL1 ubiquitination, altering disease‐associated signalling pathways, and influencing the survival or maintenance of leukaemia stem cells [24]. Previous studies have shown that bortezomib reduces SKP2 expression, promotes accumulation of p27Kip1, and enhances apoptosis in CML cells [24].

Panobinostat, an oral pan‐HDAC inhibitor used in relapsed or refractory multiple myeloma, has been shown to act synergistically with bortezomib [25]. It remains a promising, well‐tolerated agent warranting further investigation for restoring therapeutic responses in patients with disease progression after standard treatments [20]. HDAC inhibition potentiates imatinib cytotoxicity and enhances therapeutic efficacy in CML [26].

A prior clinical trial explored the combination of a proteasome inhibitor with an HDAC inhibitor in a phase I dose‐escalation study involving belinostat and bortezomib in adult patients with acute leukaemia, myelodysplastic syndromes, or blast‐crisis CML [27]. Among the 38 treated patients, the combination showed limited efficacy in relapsed or refractory AML; however, two high‐risk patients exhibited remarkable responses—one achieving complete remission and another maintaining long‐term stable disease [27].

Bortezomib and panobinostat each induced apoptosis in both TKI‐sensitive and ‐resistant CML cells, potentially through mechanisms independent of canonical BCR::ABL1 signalling. Their combination further enhanced cytotoxicity, mitochondrial dysfunction, and clonogenic suppression, highlighting dual proteasome and epigenetic inhibition as a potential approach to overcome TKI resistance. This effect is particularly pronounced in cells harbouring compound mutations resistant to most approved TKIs [28]. Mechanistic analyses revealed that the combination treatment led to substantial accumulation of ubiquitinated proteins and increased expression of pro‐apoptotic genes. Although validation using primary patient samples and normal haematopoietic cells would be important, the present study was limited to established cell line models. The use of primary human samples was not undertaken in this study because institutional ethics approval for sample collection and analysis had not been obtained at the time of study initiation. This remains an important objective for future investigations.

In conclusion, proteasome and HDAC inhibition synergistically overcome ABL TKI resistance by promoting mitochondrial apoptosis and suppressing clonogenic potential. This combinatorial approach represents a promising strategy for targeting persistent leukaemic cells that evade current TKI therapy.

## Author Contributions

Seiichi Okabe: conceptualisation (equal), formal analysis (equal), investigation (equal), writing – original draft (equal), and writing – review and editing (equal). Seiichiro Yoshizawa: methodology (equal). Yuya Arai: methodology (equal). Akihiko Gotoh: conceptualisation (equal) and supervision (equal). Daigo Akahane: conceptualisation (equal), supervision (equal), and writing – review and editing (equal).

## Funding

The authors have nothing to report.

## Conflicts of Interest

A.G. has received research support from Ono Pharmaceutical Co. Ltd.; Taiho Pharmaceutical Co. Ltd.; Chugai Pharmaceutical Co. Ltd.; Otsuka Pharmaceutical Co. Ltd.; and Asahi Kasei Co. Ltd. A.G. has also received honoraria from Novartis Pharma K.K.; Alexion Pharmaceuticals Inc.; Eisai Co. Ltd.; Ono Pharmaceutical Co. Ltd.; Taiho Pharmaceutical Co. Ltd.; Takeda Pharmaceutical Co. Ltd.; Nippon Shinyaku Co. Ltd.; Chugai Pharmaceutical Co. Ltd.; Otsuka Pharmaceutical Co. Ltd.; Sumitomo Pharma Co. Ltd.; Daiichi Sankyo Co. Ltd.; Nihon Pharmaceutical Co. Ltd.; Kyowa Kirin Co. Ltd.; Janssen Pharmaceutical K.K.; Pfizer Japan Inc.; Sanofi K.K.; and Asahi Kasei. In addition, A.G. has received consulting fees from PharmaEssentia Japan K.K.; Chugai Pharmaceutical Co. Ltd.; Alexion Pharmaceuticals Inc.; and Asahi Kasei, and has served on data safety monitoring and advisory boards for PharmaEssentia Japan K.K.; Chugai Pharmaceutical Co. Ltd.; and Alexion Pharmaceuticals Inc. S.O., S.Y., Y.A., and D.A. declare that they have no Conflicts of Interest.

## Supporting information


**S1** The effects of bortezomib and panobinostat on Ba/F3 cells.
Ba/F3 cells were treated with 10 nM bortezomib and/or 10 nM panobinostat for 72 h.
Cell viability was assessed using the CCK‐8 assay.

## Data Availability

The data that support the findings of this study are available from the corresponding author upon reasonable request.
