# Supplementary figures and images for: Dual Proteasome and Histone Deacetylase Inhibition Overcomes Tyrosine Kinase Inhibitor Resistance in Breakpoint Cluster Region: Abelson 1‐Driven Leukaemia Cell Lines
Source: J Cell Mol Med. 2026 Feb 16;30(4):e71053. doi: 10.1111/jcmm.71053 (PMC12908417; doi:10.1111/jcmm.71053)

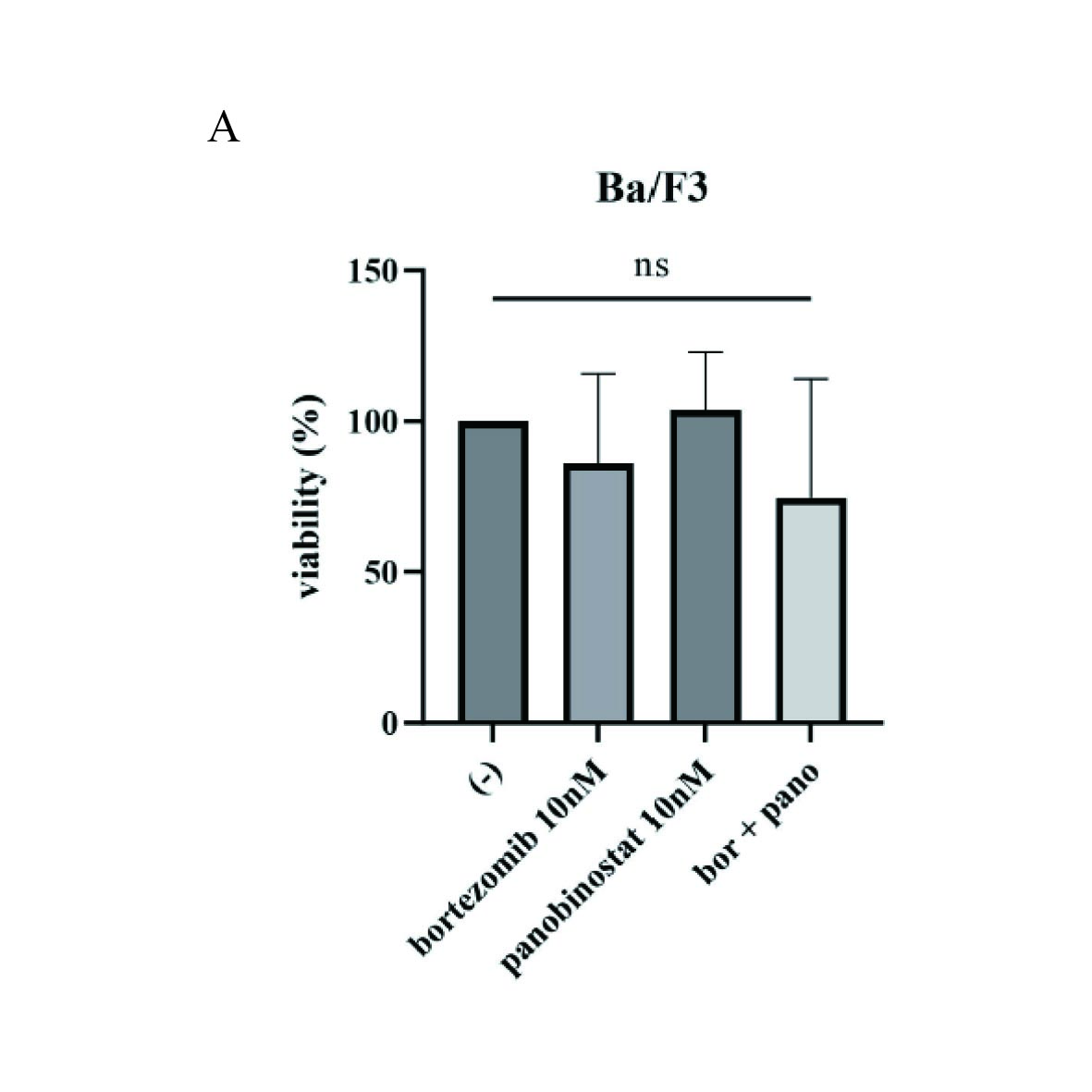

Supplement: Supplementary file 1 — S1 The effects of bortezomib and panobinostat on Ba/F3 cells. Ba/F3 cells were treated with 10 nM bortezomib and/or 10 nM panobinostat for 72 h. Cell viability was assessed using the CCK‐8 assay. [file JCMM-30-e71053-s001.jpg]
